# Neuroprotective Mechanisms and Clinical Evidence for Acupuncture in Parkinson's Disease: A Systematic Review

**DOI:** 10.1155/padi/9739567

**Published:** 2025-05-11

**Authors:** Jinting Li, Minmin Wu, Wenjing Song, Jiongliang Zhang, Luwen Zhu

**Affiliations:** ^1^Second Clinical Medical College, Heilongjiang University of Chinese Medicine, Harbin, Heilongjiang, China; ^2^The Second Hospital, Heilongjiang University of Chinese Medicine, Harbin, Heilongjiang, China

**Keywords:** acupoints, acupuncture, Parkinson's disease, RCT

## Abstract

Parkinson's disease (PD) is the second most common neurodegenerative disorder worldwide, and the mainstay of treatment is dopamine replacement therapy; however, its beneficial effects tend to wane over time as the disease progresses. Many patients seek complementary therapies to alleviate motor and nonmotor symptoms associated with PD, and the efficacy of acupuncture as a commonly used complementary and alternative therapy for PD remains controversial. In this review, we systematically examine randomized controlled trials and mechanistic studies on acupuncture for PD over the past 12 years. The findings indicate that acupuncture improves both motor and nonmotor symptoms. We also found that the mechanisms underlying the effects of acupuncture on PD may involve connectomics, modulation of dopamine and glutamate systems, regulation of the gut–brain axis, organelle homeostasis, and molecular and signaling pathway modulation. This review proposes a multitarget neuroprotection hypothesis to provide a theoretical basis for standardized acupuncture research.

## 1. Introduction

Parkinson's disease (PD) is the second most common neurodegenerative disease in the world, affecting more than 6 million people—a number projected to rise to 10 million by 2030 [1, 2]. The pathology of PD is characterized by loss and degeneration of dopaminergic neurons in the substantia nigra (SN), alpha-synuclein (α-syn) deposition, and Lewy body formation [3]. The main clinical symptoms of the disease include motor and nonmotor symptoms that severely affect the patient's quality of life and mental health [4, 5]. Despite our deep understanding of the underlying pathophysiology of PD, effective clinical interventions to alleviate patient symptoms remain lacking [6].

Acupuncture is a traditional Chinese medicine (TCM) that is commonly used to treat PD [7]. According to TCM theory, acupuncture involves selecting specific acupuncture points and applying tonic and diarrheal techniques to maintain internal balance. Acupuncture can be used to treat or relieve various diseases caused by meridian dysfunction using needling, pressure, or electrical stimulation [8–10]. In recent years, the treatment of PD with acupuncture has been widely investigated in basic and clinical studies, and animal experiments have demonstrated that acupuncture can have a volatile neuroprotective effect. Clinical randomized controlled trials (RCTs) have also reported that acupuncture can improve motor and nonmotor symptoms. However, the heterogeneity of acupuncture protocols and the lack of uniformity in efficacy evaluation criteria have limited the level of evidence. Mechanistic studies have focused on individual pathways, lacking systematic integration at the multiomics level. These challenges hinder the standardized application of acupuncture therapy and its inclusion in international treatment guidelines.

This paper systematically reviews RCTs and mechanistic studies on acupuncture for PD, summarizes its effectiveness in treating patients with the disease, and explores its multitarget mechanisms to strengthen the evidence supporting acupuncture as a treatment for PD.

## 2. Methods

### 2.1. Search Strategy

We searched four international electronic databases (PubMed, Embase, Cochrane Library, and Web of Science databases) and one Chinese electronic database, the China National Knowledge Infrastructure (CNKI). The inclusion of CNKI aimed to supplement high-quality clinical and basic research on acupuncture published in Chinese journals that may not be indexed internationally, ensuring comprehensive evidence access. The searches were limited to the past 12 years (March 2013 to March 2025). The search terms and basic strategy were as follows: (Acupuncture term) AND (Parkinson term) AND (clinical trial OR mechanism study). Detailed search terms and strategies are listed in Supporting Information Methods S1.

### 2.2. Inclusion and Exclusion Criteria

Studies were included if they met the following criteria. They had to be RCTs involving patients diagnosed with PD and its complications [11, 12], with no restrictions on age, sex, race, treatment, or disease duration. The intervention had to involve acupuncture, including electroacupuncture, head acupuncture, auricular acupuncture, head acupuncture, or body acupuncture, either as the primary treatment or in combination with conventional treatment. The control group design included a sham acupuncture group, conventional drug group, blank control, or other nonacupuncture interventions.

Studies were excluded if they involved incompatible study types, including animal experiments, in vitro studies, case reports, conference abstracts, reviews, and nonsystematic reviews. Incompatible interventions included studies where acupuncture was used only as an adjunctive treatment and its separate effects could not be isolated, as well as traditional therapies other than acupuncture (e.g., herbal medicine alone or tuina). Additionally, studies with incomplete data, where too little information was included or the data were unavailable, were also excluded. Finally, duplicate reports of the same study were excluded.

### 2.3. Literature Screening and Data Extraction

Literature was managed and screened using the Zotero literature management tool. Two evaluators independently screened studies according to the inclusion and exclusion criteria, and the titles and abstracts of the included studies were evaluated. If there was any doubt regarding the inclusion of a study, the full text of the article was retrieved. Any disagreements regarding the inclusion were resolved through discussion or consultation with a third reviewer.

After selecting the studies based on the inclusion and exclusion criteria, data were extracted independently by two evaluators using a self-developed data extraction table. The specific features extracted included the first author, year of publication, number of included RCTs, number of participants, details of the acupuncture intervention, type of control, quality evaluation tool of the RCTs, outcome measures, method of data synthesis, main results, and conclusions. If the existing information was unclear or lost, the corresponding author of the study was contacted via email.

### 2.4. Quality Assessment

Two reviewers independently assessed the quality of the included RCTs using the revised Cochrane Risk of Bias Tool Version 2 (RoB2) [13]. Where disagreements arose, they were discussed with another researcher. The randomization process, bias between the intended and delivered interventions, data completeness, and outcome measures were carefully assessed for each study and subsequently classified as having low, high, or unclear potential risk or bias.

## 3. Results

### 3.1. Search Results

**Figure 1:**
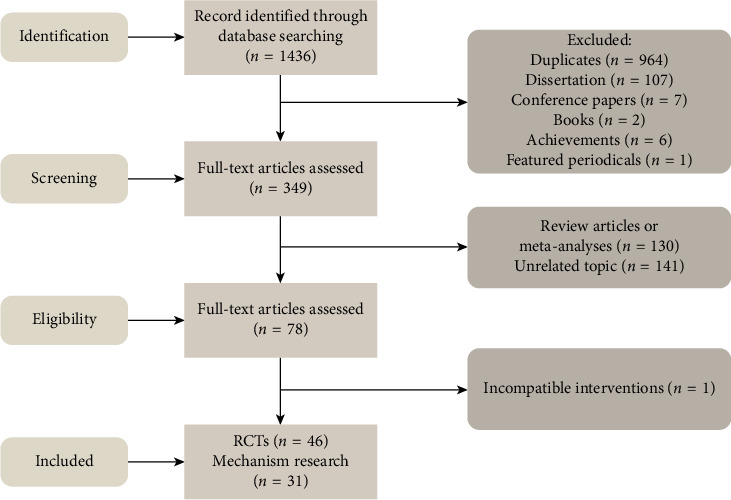
Flow diagram regarding the article selection for the overview.

A computerized database search yielded 1436 references. After reading the titles and abstracts, and excluding reviews and literature unrelated to the topic, 46 RCTs and 31 mechanistic studies were included. The process and results of the literature screening are presented in Figure 1.

### 3.2. Quality Evaluation of Included Studies

**Figure 2:**
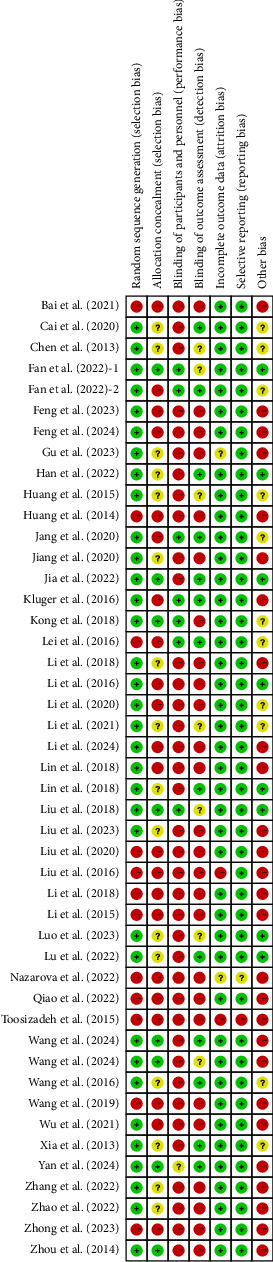
Risk of bias summary.

**Figure 3:**
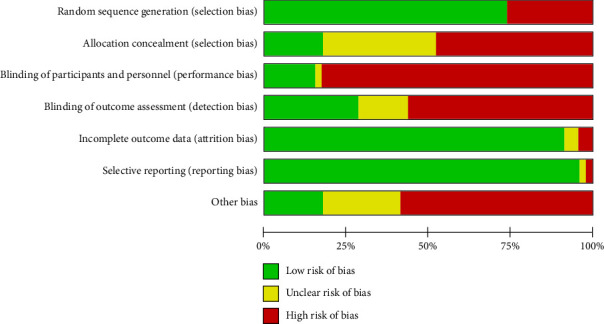
Risk of bias graph.

We assessed the risk of bias in all included studies. The detailed bias is summarized in Figures 2 and 3 and Table S1.

### 3.3. Clinical Studies of Acupuncture in the Treatment of PD

Acupuncture can be used to treat or alleviate various diseases caused by meridian dysfunction using needling, pressure, or electrical stimulation. RCTs of acupuncture for the treatment of patients with PD have shown its efficacy for motor and nonmotor symptoms, which has prompted further evaluation (Table 1).

#### 3.3.1. Efficacy of Acupuncture in the Treatment of PD

We observed synergistic effects of acupuncture in an RCT summarizing acupuncture treatment for PD. Two RCTs [17, 24] found that, compared to medication alone, acupuncture intervention significantly reduced the Modified Webster Scale score in patients with PD, with a higher overall effectiveness rate than that of the monotherapy group. Several studies [28, 36, 37, 39, 42, 45] have shown that the acupuncture combined with medication group had lower Unified Parkinson's Disease Rating Scale (UPDRS) scores (*p* < 0.05) and improved activities of daily living scores compared to the medication or acupuncture alone group.

#### 3.3.2. Acupuncture for Motor Dysfunction

Several RCTs have confirmed that acupuncture combined with conventional treatment significantly improves motor function and gait impairment in patients with PD. Han et al. [25] showed that acupuncture combined with conventional treatment (medication and rehabilitation training) resulted in lower UPDRS-III (motor function) and UPDRS-IV (motor complication) scores after treating patients with PD than in the conventional treatment group. Liu and Wu [30] further showed that the decrease in the frequency and amplitude of tremor muscle potentials was more pronounced with acupuncture combined with drugs than with conventional treatment of tremors, suggesting an advantage of acupuncture in the modulation of tremor symptoms.

In terms of balance function, Toosizadeh et al. [57] showed that electroacupuncture combined with drug treatment for 8 weeks resulted in a 31% reduction in the lateral center of gravity sway (COGML/AP) and a 46% improvement in ankle-hip coordination in patients with PD, suggesting that acupuncture improves postural stability and motor coordination using three-dimensional motion analysis. Feng et al. [35] showed that the acupuncture combined treatment group had higher Berg Balance Scale (BBS) scores and lower UPDRS-III scores than the drug-only group.

In terms of muscle stiffness, Wang et al. [14] showed that 100 Hz high-frequency electroacupuncture combined with medication significantly outperformed the 2/100 Hz electroacupuncture and medication-only groups in improving myotonia in patients with PD during long-term follow-up, with a decrease in UPDRS-III and Tone Assessment Scale scores in the intervention group compared with baseline, and with a continued reinforcement of efficacy over time. Fan et al. [19] further reported that after 8 weeks of governor three needles combined with hand and foot contracture three-needle intervention, the test group showed a decrease in Ashworth Spasticity Scale scores, an increase in 10-m walking speed, and an increase in brain-derived neurotrophic factor (BDNF) levels from baseline. This study confirmed that acupuncture may improve muscle stiffness and motor coordination by activating the endogenous BDNF signaling pathway.

In terms of gait, Zhong et al. [44] and Li et al. [23] showed that the combined acupuncture and moxibustion treatment group elevated gait speed, increased stride length, and shortened 20-m walking time. Lei et al. [53] showed a significant elevation of gait speed after electroacupuncture intervention, an increase in stride length in dual-task rapid walking, and an improvement in angular velocity in the mid-swing by multitasking gait analysis. Jang et al. [51] utilized the GAITRite system in conjunction with the fNIRS technique and confirmed that acupuncture significantly increased stride length and prefrontal cortex oxygenated hemoglobin levels. Furthermore, oxygenated signals were positively correlated with gait cycle parameters, suggesting that acupuncture may improve motor control through cortical neural remodeling.

In conclusion, acupuncture combined with conventional treatment synergistically improved PD motor symptoms, optimized gait parameters, and enhanced balance, thereby providing evidence-based support for the treatment of PD.

#### 3.3.3. Acupuncture for Nonmotor Dysfunction

Several RCTs [21, 26, 31, 43] have shown that acupuncture combined with conventional treatment significantly improves anxiety–depression status in patients with PD. Xia et al. [18] showed that electroacupuncture effectively regulated serum DA concentrations in patients with PD complicated by depression, thus alleviating depressive symptoms. Fan et al. [50] showed that after 8 weeks of acupuncture treatment, patients' HAM-A scores decreased, and serum adrenocorticotropic hormone and cortisol levels were reduced. Acupuncture may improve the overall motor function and well-being of patients with PD by alleviating anxiety.

For PD-mild cognitive impairment (PD-MCI), two RCTs [15, 29] demonstrated that acupuncture combined with medication improved the Montreal Cognitive Assessment Scale and Mini-Mental State Examination scores and improved cognitive functioning in patients with PD-MCI.

Multiple RCTs [22, 27, 40, 49, 59] have assessed sleep quality in treated patients. In these trials, decreased Pittsburgh Sleep Quality Index scores and increased Parkinson's Disease Sleepiness Scale (PDSS) scores were observed after acupuncture was combined with medication. Li et al. [16] found that 5-HT, DA, and BDNF levels were elevated in the acupuncture combined drug group compared to those in the control group, and the degree of elevation was more pronounced in the combined group than in the Western drug group. Another RCT [56] found reduced Non-Motor Symptom Scale (NMSS) sleep/fatigue subscale scores, and PDSS and UPDRS scores after an 8-week course of cephaloabdominal electroacupuncture. Additionally, 16S rRNA sequencing revealed that the relative abundances of the genera *Mycobacterium similis* (*Bacteroides*) and *Parasutterella* were significantly increased after the intervention, whereas those of *Mycobacterium minutissimus*, *Hungatella*, *Barnesiella*, *Megalococcus*, *Allisonella*, *Intestinimon*, and *Moryella* were significantly reduced. The abundance of short-chain fatty acid–producing genera (e.g., Parasutterella) was positively correlated with serum BDNF levels. This suggests that acupuncture can be used as a complementary alternative therapy to improve serum levels and sleep quality in patients, suggesting a potential role of the gut–brain axis in this process.

#### 3.3.4. Acupuncture for Wearing-Off Phenomenon

Jia et al. [33] found that LDE scores were lower in the acupuncture group than in the drug group and that the pre- and post-treatment dose differences were lower in the acupuncture group than in the drug group. Lin et al. [41] further confirmed that the incidence of end-of-dose wearing-off was significantly decreased in the acupuncture combined with the conventional treatment group compared with the conventional drug group. This suggests that acupuncture combined with medication has a higher efficacy rate in the treatment of PD and reduces the dosage of Western medication, which has the potential advantage of reducing toxicity and increasing efficacy.

#### 3.3.5. Acupuncture for Constipation

Several RCTs [20, 47, 48] have evaluated the effects of acupuncture on constipation in patients with PD. Conventional Western medicine was used as the control group, and the treatment group was combined with acupuncture intervention. The assessment indices included anorectal dynamics (rectal resting pressure and defecation threshold), constipation symptom score, quality of life scale (PAC-QOL and SF-36), and autonomic function (SCOPA-AUT). The results showed that the acupuncture group significantly outperformed the Western medicine-only group in improving core constipation symptoms, such as defecation frequency and fecal character, as well as in elevating rectal and anal canal pressures and lowering the defecation threshold. Additionally, acupuncture enhanced autonomic function, suggesting its ameliorative effect on constipation symptoms in patients with PD, which is worthy of clinical promotion.

#### 3.3.6. Acupuncture for Swallowing Disorders

Two RCTs [32, 34] found that acupuncture combined with conventional treatment improved dysphagia in patients with PD compared to conventional treatment. Feng et al. [32] randomly divided 60 patients with PD into an acupuncture–medicine combination group (conventional medication + rehabilitation training + acupuncture intervention) and a control group, with acupuncture targeting the Fengfu, Baihui, and pharyngeal triple acupuncture points for intervention. The results showed that the Standardized Swallowing Function Scale score decreased, and the Swallowing-related Quality of Life Scale score increased, which was significantly better than that of the control group. Wu et al. [34] further quantitatively assessed using videofluoroscopic swallowing function screening and found that the needle and medicine groups had shorter oropharyngeal transfer time and reduced laryngeal closure time than the control group, along with higher serum albumin levels. The total effective rate of the combined group was significantly higher than that of the control group, and the difference was statistically significant in all cases. This suggests that acupuncture improves local blood circulation by activating glossopharyngeal nerve endings, thus enhancing swallowing efficiency and nutritional status.

#### 3.3.7. Acupuncture for Upright Hypotension

Li et al. [38] evaluated the efficacy of stellate ganglion acupuncture combined with drug therapy in an RCT. The results showed that standing systolic blood pressure and diastolic blood pressure increased by 23 and 12 mmHg, respectively, in the acupuncture-medicine combination group after the intervention compared with baseline, significantly outperforming the Western medicine group alone. Serum norepinephrine (NE) levels were higher in the combination group than in the Western medicine group. In terms of clinical efficacy, the combination group showed a 52% reduction in the upright hypotension questionnaire score and a decrease in the UPDRS score, and the overall efficacy rate was higher than that of the Western medicine group. These findings provide evidence that acupuncture modulates upright hypotension and improves the complications of PD.

#### 3.3.8. Acupuncture for Fatigue

As patients with PD often include fatigue in their symptoms, whether acupuncture can improve fatigue is also a question that needs to be explored. Liu et al. [46] conducted an RCT of 120 patients in which acupuncture combined with conventional treatment (targeting acupoints such as Foot Sanli and Sanyinjiao) for 4 weeks resulted in a reduction in Fatigue Severity Scale scores, and the efficacy was sustained for up to 3 months. However, studies by Kong [52] and Kluger [54] used sham acupuncture controls, which included 40 and 94 patients, respectively, for 5–6 weeks. The results showed significant improvements in fatigue scales (MFIS and MFIS-Total) and quality of life (PDQ-39) in both the real and sham acupuncture groups. However, there was no statistically significant difference between the groups, suggesting that the efficacy may be a placebo effect or a nonspecific mechanism.

#### 3.3.9. Acupuncture for Pain Improvement

Shaosong et al. [55] evaluated electroacupuncture as an intervention for skeletal muscle pain in PD through an RCT. After 4 weeks of high-frequency electroacupuncture intervention, the electroacupuncture group, compared to the control group, showed not only the usual effects but also a decrease in pain relief, as indicated by lower scores on the King's Parkinson's Pain Scale (KPPS) and the Visual Analog Scale (VAS). Another study [58] used fire needle therapy and found that the FNT group showed a decrease in KPPS and VAS scores at week eight of chronic pain management compared to the control group. These findings suggest that acupuncture is an effective and safe treatment option for chronic PD-related pain (Table 2).

### 3.4. Mechanisms of Acupuncture for PD

#### 3.4.1. Brain Network Modulation Mechanisms in PD

Connectomics, a tool for detecting perturbations in structural and functional networks in neurodegenerative diseases through neuroactive markers (e.g., c-Fos), provides an important pathway for resolving networks across brain regions in neurodegenerative diseases [91]. The characteristic pathological changes in PD are not limited to the substantia nigra pars compacta (SNpc )–striatal dopaminergic pathway but also involve synaptic connections in several brain regions [92, 93]. Acupuncture stimulation at point GB34 bilaterally in MPTP mice demonstrated that acupuncture improved motor dysfunction, prevented disruption of dopaminergic nerve fibers, and increased the number and strength of associations between brain regions following MPTP treatment in PD mice. Brain connectivity analysis showed that stimulation at point GB34 restored c-Fos activity in the primary motor area (M1) and significantly restored neuronal activity in other brain regions normally associated with motor behavior (striatum, SNpc, and pallidum), as well as nonmotor symptoms (hippocampus (HPC), lateral hypothalamus, and isolated brain regions) [75].

#### 3.4.2. Dopamine and Glutamate Systems

##### 3.4.2.1. Regulation of Dopamine Receptors and Transporters

PD is caused by extensive damage to the dopamine-producing SN neurons, leading to midbrain dopamine deficiency and subsequent changes in other neurotransmitters [94]. In 6-hydroxydopamine (6-OHDA) rats, 100 Hz electroacupuncture at SP6, GB34, and ST36 restored dopamine D1 receptor expression, prevented the upregulation of dopamine D2 receptor, and attenuated motor dysfunction [79]. This suggests that electroacupuncture at SP6, GB34, and ST36 can act on presynaptic dopamine transporters and postsynaptic dopamine receptors to achieve therapeutic efficacy in the treatment of PD, which is one of the potential mechanisms of acupuncture in the treatment of PD.

##### 3.4.2.2. Dynamic Regulation of Glutamatergic Systems

###### 3.4.2.2.1. Functional Regulation of Vesicular Transporter Proteins

Vesicular glutamate transporter protein 1 (VGluT1) is one of the vesicular transporter proteins responsible for glutamate release. In 6-OHDA-impaired hemiparkinsonian rats, 100-Hz electrical stimulation at GV20 and GV14 upregulated the expression of VGluT1 in the subthalamic nucleus, thereby alleviating motor symptoms [71].

###### 3.4.2.2.2. Synaptic Plasticity Repair

In a rat model of unilateral 6-OHDA damage, loss of striatal functional synaptic plasticity was observed, including long-term potentiation (LTP) and long-time-range depression. Structural striatal synaptic plasticity is also impaired, with reduced dendritic branching and decreased spinal density. In unilaterally injured 6-OHDA rats, transcutaneous electrical stimulation at GV20 and GV14 enhanced striatal LTP and upregulated the expression of N-methyl-D-aspartate receptor subunit NR2B. The intervention also increased dendritic branching and spine density in the striatum, suppressed glutamate and VGluT1 levels, reversed the loss of spine density, and attenuated dyskinesia [72]. It was further found that electroacupuncture inhibited the overtransmission of cortical striatal glutamatergic synapses, reduced striatal glutamate levels, reversed abnormal synaptic plasticity (e.g., dendritic spine density, vGluT1+ terminal density), and effectively suppressed abnormal cortical striatal synchronized high-beta (25–40 Hz) oscillations, which can be used as a pathological biomarker of PD [87].

###### 3.4.2.2.3. Metabotropic Glutamate Receptor Activation

A significant reduction in mGluR2/3 protein expression and mGluR3 mRNA levels has been observed in the corticostriatal pathway in PD model animals [95]. Following prolonged 100-Hz electrical stimulation at GV20 and GV14 in a mouse model of 6-OHDA injury, Group II mGluRs were activated, and mGluR2/3 activity was enhanced, which would automatically inhibit presynaptic glutamate release and reduce extracellular glutamate levels. Furthermore, activation of mGluR3 enhanced the production of several neurotrophic factors, including transforming growth factor-β and glial cell line–derived neurotrophic factor (GDNF), thereby ameliorating motor deficits in the mouse model [73].

###### 3.4.2.2.4. Reconstruction of Glutamate Metabolic Homeostasis

In rats with unilateral 6-OHDA administration, the ratio of glutamate and glutamine (Glx) to creatine (Cr) significantly increased in the striatum. Simultaneous electroacupuncture stimulation at GV20 and GV14 reversed the imbalance in the Glx/Cr ratio in the bilateral striatum and improved locomotor performance in rats [74].

We suggest that acupuncture can modulate glutamatergic transmission through (1) dynamic regulation of vesicular transporter proteins, (2) synaptic plasticity repair, (3) metabotropic glutamate receptor activation, and (4) glutamatergic metabolic homeostasis, providing a theoretical rationale for a nondopaminergic therapeutic strategy for modulating the glutamatergic system.

#### 3.4.3. Regulation of the Gut–Brain Axis (GBA)

The GBA is an important bridge connecting the enteric nervous system (ENS) and the central nervous system, and it regulates homeostasis in concert with the intestinal microbiota through neuro-immuno-endocrine pathways [96]. The modulation of the GBA by acupuncture plays an important role in the treatment of PD, and its mechanism involves the following aspects.

##### 3.4.3.1. Regulation of Inflammation in the Central and Peripheral Nerves

Gastrointestinal inflammation is an early nonmotor symptom of PD and is closely associated with the degeneration of dopaminergic neurons in the SN. Acupuncture at CV12 (midcavity), CV4 (coronal), and ST25 (sky pillar) in 6-OHDA rats significantly reduced serum and duodenal proinflammatory factors (IL-1β, TNF-α, and IFN-γ) levels. Additionally, it significantly decreased iron deposition in the nigral region by regulating the expression of iron metabolism-related proteins. By balancing the expression of divalent metal transporter 1 and ferroportin 1, a protein related to iron metabolism, it can reduce the iron deposition in the SN [77].

##### 3.4.3.2. Regulation of Gut Microbiota–Metabolites

The gut microbiota and its metabolites are involved in the pathology of PD through the microbe–GBA [97, 98]. Jang et al. [60] showed that acupuncture at the GB34 and ST36 acupoints significantly improved motor function and anxiety behaviors, and reduced dopaminergic neuron loss in the SN and striatum in an MPTP-induced mouse model of PD. Additionally, 16S rRNA sequencing showed that acupuncture reversed the trend of decreased alpha diversity in the gut flora of PD mice, significantly increasing the number of butyric acid–producing genera of *Bacteroidetes* (e.g., *Butyricimonas*) and decreasing the proinflammatory phylum Proteobacteria (e.g., *Holdemania*). Han et al. [61] compared the effects of electroacupuncture (ST36 and GV20) with those of hand acupuncture in a mouse model of MPTP-induced PD. The results showed that electroacupuncture significantly improved motor deficits and restored the number of nigrostriatal dopaminergic neurons. Gut microbiota analysis showed that the abundance of Erysipelotrichaceae was significantly elevated in PD mice, whereas its abundance was reduced after electroacupuncture intervention, and alpha diversity (Simpson's index) was restored to normal levels. Meanwhile, electroacupuncture downregulated the decrease of IL-6 and TNF-α mRNA expression, suggesting that it ameliorated the pathology of PD by inhibiting the inflammatory response mediated by gut microbiota dysregulation. Hu et al. [83] used a rat model of rotenone-induced PD and found that electroacupuncture at ST25 acupoints significantly improved motor function and protected dopaminergic neurons in the SNpc. Analyses of 16S rRNA and metabolomics showed that electroacupuncture increased beneficial bacterial genera (*Lactobacillus* and *Bifidobacterium*), decreased pathogenic genera (*Escherichia-Shigella* and *Morganella*), attenuated lipopolysaccharide (LPS)-induced inflammatory response and oxidative stress, and improved motor dysfunction.

##### 3.4.3.3. Repair of the Gut Barrier

Gut barrier permeability is correlated with tight junction proteins in the gastrointestinal epithelium, including occludin, gap junction proteins, and zona occludens (ZO) [99]. Electroacupuncture stimulation at DU24, ST25, ST37 (upper giant vacuole), and LI11 in rotenone-induced mice increased the expression levels of ZO-1 and occludin, decreased permeability, and attenuated dyskinesia and gastrointestinal inflammation [82]. In MPTP mice, electroacupuncture stimulation at GV16, LR3, and ST36 resulted in increased claudin-1 expression in the colon, decreased activation of NLRP3 inflammatory vesicles, improved intestinal barrier integrity, suppression of inflammatory cytokine activation in the colon, and attenuation of behavioral deficits, which exerted a protective effect against PD [85].

##### 3.4.3.4. Brain–Gut Peptide Regulation

Brain–gut peptides (e.g., NPY, CCK, SST, GAS, and PYY) exert bidirectional regulation between the gastrointestinal and central regions [100, 101]. Electroacupuncture stimulation at CV12, CV6 (Qihai), LR3, and ST36 in 6-OHDA-induced rats upregulated serum and SN levels of NPY, CCK, SST, and GAS, while decreasing the expression of the proapoptotic peptide PYY, thereby alleviating PD symptoms [62]. Among them, NPY attenuates neuroinflammation by inhibiting microglial activation, CCK improves motor function by modulating synaptic plasticity in dopaminergic neurons, and GAS restoration correlates with gastrointestinal dyskinesia remission [102–106].

In summary, we believe that acupuncture at gastrointestinal-related acupoints such as CV12, CV4, and ST25: (1) inhibits central and peripheral inflammatory responses, (2) regulates intestinal flora–metabolite profiles, (3) repairs the integrity of the intestinal barrier, and (4) regulates the brain–gut peptide balance. Future research should focus on analyzing specific flora–metabolite–neuroimmune pathways and conducting clinical translational studies to verify their efficacy.

#### 3.4.4. Organelle Homeostasis Regulation

##### 3.4.4.1. Maintain Endoplasmic Reticulum Homeostasis

Homeostatic imbalance in the endoplasmic reticulum, a central site for protein synthesis, folding, and modification, can trigger the unfolded protein response (UPR), in which the inositol-requiring enzyme 1 (IRE1) signaling pathway plays a key role. The endoplasmic reticulum plays a key role in the UPR. In the PD model, the accumulation of aberrant proteins leads to the activation of IRE1 phosphorylation, which in turn shears X-box binding protein 1 (XBP1) to enhance molecular chaperone expression and promote misfolded protein degradation [107, 108]. Acupuncture stimulation at LR3, LI4, GV20, GB20, DU16, and ST36 in 6-OHDA rats significantly upregulated the expression levels of phosphorylated IRE1 (p-IRE1) and XBP1, restored the endoplasmic reticulum's protein quality control ability, reduced the abnormal aggregation of midbrain α-synaptic nuclear proteins, and elevated the striatal dopamine and serotonin levels, which resulted in improved motor coordination and sensory integration function in PD model rats. This suggests that acupuncture can maintain endoplasmic reticulum homeostasis and correctly process, modify, and fold proteins through the IRE1/XBP1 pathway [76].

##### 3.4.4.2. Repair of Mitochondrial Dysfunction

Mitochondria, which are the core organelles involved in the regulation of energy metabolism and autophagy, are often dysfunctional in PD. In MPTP-induced PD mice, the expression levels of parkin, PTEN-inducible kinase 1 (PINK1), peroxisome proliferator-activated receptor γ coactivator 1-α, and mitochondrial fusion protein 2 (Mfn2) were significantly downregulated, whereas the expression level of dynamin-associated protein 1 (Drp-1) was significantly upregulated, resulting in a decrease in mitochondrial membrane potential. Electroacupuncture stimulation of the tremor control region in MPTP mice activated transient receptor potential channel 1 (TRPC1) channels and regulated calcium homeostasis to maintain mitochondrial membrane potential. It enhanced mitochondrial biosynthesis through the deacetylase 1 (SIRT1)/AMP-activated protein kinase (AMPK) pathway, upregulated the expression of PGC-1α and Mfn2, and inhibited Drp1-mediated hyperdissection. This synergistic effect not only restored mitochondrial energy metabolism but also promoted the clearance of damaged mitochondria through autophagy, thereby blocking the degenerative process of dopaminergic neurons [81].

The above findings systematically elucidate the molecular basis of acupuncture intervention in the pathological process of PD through a multidimensional organelle homeostasis regulatory network and provide new ideas for optimizing neuroprotective strategies in PD.

#### 3.4.5. Molecular and Cellular Signaling Pathways

##### 3.4.5.1. Regulating Autophagy

Autophagy, a major clearance mechanism for intracellular protein aggregates and damaged organelles, is essential for maintaining neuronal homeostasis by degrading aberrant proteins (e.g., α-synuclein) and mitochondria via the autophagosome–lysosome pathway [109–112]. Acupuncture may improve the pathological progression of PD by modulating the autophagic process; however, the direction and mechanism of action are controversial. In MPTP mice, pinprick stimulation of GB34 enhanced autophagic clearance of α-synuclein, restored mitochondrial and lysosomal structure, and ameliorated symptoms of PD [63]. Zhang et al. [88] used wild-type (WT) and nuclear factor erythroid 2–related factor 2 (Nrf2) knockout (KO) mice to construct an MPTP model. After receiving electroacupuncture, WT mice showed an increase in TH-positive neurons, a decrease in α-synaptic nuclear proteins, an upregulation of autophagy markers Atg7 and LC3II, a reduction in p62 levels, an increase in autophagosomes and lysosomes, elevated Nrf2 expression in the nucleus, and inhibition of Keap1. However, the neuroprotective effects and autophagy regulation of electroacupuncture were completely lost in KO mice. Conversely, in 6-OHDA rats treated with acupuncture at GV20, LI4, LR3, and DU4, beclin-1 expression was downregulated, while p62 expression was upregulated. Subsequently, this reduced the number of autophagosomes and activated mammalian rapamycin-targeted protein mTOR pathway, which repaired damaged synapses by inhibiting the autophagic process [64].

In summary, autophagy regulation by acupuncture is bidirectional. On the one hand, it ameliorates MPTP-induced PD pathology by activating the Nrf2 signaling pathway, restoring autophagic flow, and removing α-synuclein. On the other hand, it activates the mTOR pathway and inhibited autophagy to promote synaptic repair.

##### 3.4.5.2. Inhibition of Apoptosis

Apoptosis is a form of programmed cell death that effectively builds neural networks in the brain; however, excessive apoptosis accelerates the progression of PD [113]. In MPTP mice, acupuncture at GB34 and ST36 enhanced the expression of B-cell lymphoma 2 (BCL-2) while suppressing that of BCL-2-associated X protein, thereby inhibiting apoptosis [60]. In 1-methyl-4-phenylpyridine (MPP+) rats, electroacupuncture stimulation at GB34 and LR3 increased BCL-2 expression, which attenuated motor nerve activity and turning behavior [84]. Caspase-3 is a cysteine–aspartate protease that, when activated, cleaves various downstream substrates during apoptosis, leading to the morphological changes characteristic of apoptotic cells [114]. In MPTP mice, acupuncture at GB34 and SI3 (backstream) significantly reduced striatal caspase-3 levels, thereby inhibiting apoptosis [65]. Therefore, acupuncture can inhibit apoptosis, protect neuronal cells, and restore motor function, making it a promising clinical treatment for PD.

##### 3.4.5.3. Neuroinflammation and Oxidative Stress

Neuroinflammation and oxidative stress usually characterize PD pathology, whereas abnormal microglial activation-promoting factor–redox homeostasis imbalance accelerates dopaminergic neuronal degeneration [115, 116]. Acupuncture exerts a neuroprotective effect through the multitargeted modulation of this pathological axis, and its mechanism of action is categorized as follows:

###### 3.4.5.3.1. Anti-Inflammatory and Antioxidant Coregulation Mechanisms

Electroacupuncture stimulation at ST36 and SP6 in the A53Tα-synaptic nuclei reduced the activation level of microglia, inhibited the abnormal increase of proinflammatory factors such as TNF-α and IL-1β in the striatum and midbrain, and prevented the loss of dopaminergic neurons and motor neurons in the spinal cord and midbrain [66]. This is closely related to the activation of the Nrf2 signaling pathway. Electroacupuncture intervention blocked NF-κB-mediated neuroinflammatory signaling by upregulating the expression of Nrf2 and its downstream antioxidant response element target genes (e.g., HO-1, NQO1) [67].

###### 3.4.5.3.2. Oxidative Stress Regulatory Pathways

In MPTP mice, acupuncture at GB34 increased expression of PD protein 7 (DJ-1) in the striatum, which directly regulates superoxide dismutase and catalase, restoring their activity and exerting antioxidant effects [65]. Additionally, serum/glucocorticoid-regulated kinase 1 (SGK1), which is activated in response to glucocorticoid and serum stimulation, is thought to induce antioxidant activity [117]. MPTP induced a decrease in SGK1 expression in mice, whereas acupuncture at GB34 and LR3 upregulated SGK1 expression in the SN and alleviated PD symptoms via an antioxidant mechanism [78].

###### 3.4.5.3.3. Regulation of Ion Channels

Electroacupuncture stimulation at the tremor control region in the head of PD mice exerted neuroprotective effects by significantly upregulating nigrostriatal dopaminergic neuronal markers, including tyrosine hydroxylase (TH), PARKIN, and PINK1, while inhibiting α-syn expression. At the level of immune modulation, it inhibited M1 phenotypic markers (iNOS and CD86) and promoted the expression of M2 repair phenotypic markers (Arg-1 and CD206). Electroacupuncture activated TRPC1, elevating intracellular Ca^2+^ concentration, and downregulated SGLT1 expression to establish the TRPC1/Ca^2+^→SGLT1 axis. This mechanism synergistically inhibited neuroinflammation and oxidative stress [89]. Notably, this study was the first to elucidate that electroacupuncture alleviates PD through a dual role of metabolic reprogramming and immune regulation. It proposed SGLT1 and TRPC1 as new targets for nondopaminergic therapy, providing a theoretical basis for the treatment of PD with acupuncture. Further exploration is needed to fully understand the clinical translational potential and the mechanism of whole-brain metabolic network regulation.

##### 3.4.5.4. Neurotrophic Factors and Neuropeptides

Neurotrophic factors, such as BDNF and GDNF, along with neuropeptides like melanin-concentrating hormone (MCH), form core networks that regulate dopaminergic neurons [118–121]. Electroacupuncture stimulation at GV16 and LR3 upregulates the mRNA expression of brain BDNF and GDNF, which increase neurotrophic factor levels and improve motor dysfunction by enhancing TH activity [68]. Similarly, electroacupuncture stimulation at GV20 and GV14 (great vertebrae) had the same effect [69]. In addition, electroacupuncture stimulation at GV20 and GV29 reversed the imbalance between full-length (TrkB FL) and truncated (TrkB T1) troponin receptor kinase B (TrkB) isoforms, restored the activation of the BDNF-TrkB signaling pathway, and increased the expression of BDNF to improve motor dysfunction [70]. In an MPP + rat model, electroacupuncture at GB34 and LR3 activated survival pathways involving protein kinase B and BDNF in the SN, increasing BDNF expression and alleviating motor dysfunction [84]. Acupuncture stimulation at GB34 in A53T mutant mice significantly increases MCH expression in the lateral hypothalamus, increases MCH levels in the SN, and alleviates motor dysfunction in PD [80].

In summary, acupuncture promotes neurotrophic factors and neuropeptides, maintaining the homeostasis of dopaminergic neurons.

#### 3.4.6. Other Mechanisms

##### 3.4.6.1. Mechanisms of Mast Cell (MC)–Mediated Regulation of Peripheral Immunity

Oh et al. [90] showed that acupuncture stimulation of the GB34 acupoint significantly increased the number and degranulation ratio of local MCs, released mediators such as histamine decarboxylase and tryptase-like enzymes, which improved motor symptoms and anxiety-like behaviors, restored dopaminergic neuron activity in the SN and striatum, and inhibited neuroinflammatory markers (IL-6, IL-1β, and TNF-α) and glial cell activation. However, pretreatment with the MC stabilizer cromolyn blocked these therapeutic effects, whereas the local anesthetic lidocaine inhibited symptomatic improvement but did not affect MC degranulation, suggesting that MC activation is an upstream mechanism of acupuncture effects. Acupuncture further promotes MC activation by altering the collagen fiber morphology at acupoints through mechanical stimulation. This suggests a central role for peripheral MCs in the treatment of PD with acupuncture and provides a new basis for the regulation of neuroimmune pathways by acupuncture.

##### 3.4.6.2. Mechanisms of Neural Circuits Mediated by Hypothalamic MCH Neurons

Oh et al. [86] showed that acupuncture stimulation at the GB34 acupoint in the hind limbs of PD model mice activates hypothalamic MCH neurons via peripheral nerve conduction. Through chemical genetic manipulation and viral tracer techniques, it was clarified for the first time that MCH neurons consist of two anatomically and functionally independent subpopulations. MCH^LH/ZI→SNpc^ neurons projecting to the SNpc are mainly responsible for ameliorating motor dysfunctions, whereas MCH^LH→HPC^ neurons projecting to the HPC specifically mediate the recovery of memory functions. Inhibition of MCH neurons blocked the ameliorative effects of acupuncture on motor retardation, dopaminergic neuron loss, and gliosis in PD mice, whereas the activation of MCH neurons mimicked the efficacy of acupuncture. Activation of the MCH-MCHR1 signaling pathway mediates the protective effects of dopaminergic neurons, reduces glial cell proliferation, and enhances synaptic plasticity in the HPC. Transcriptome analysis showed that both acupuncture and MCH activation reversed PD-related gene expression abnormalities, elucidating the mechanism by which acupuncture treats PD through the hypothalamic MCH neural circuits.

## 4. Discussion

Systematic exploration of the clinical efficacy and mechanism of action of acupuncture as a multitargeted intervention strategy for PD has provided a new scientific perspective for the treatment of PD with nonpharmacological therapies. However, the complexity of the mechanism and the heterogeneity of the efficacy still need to be cautiously analyzed.

**Figure 4:**
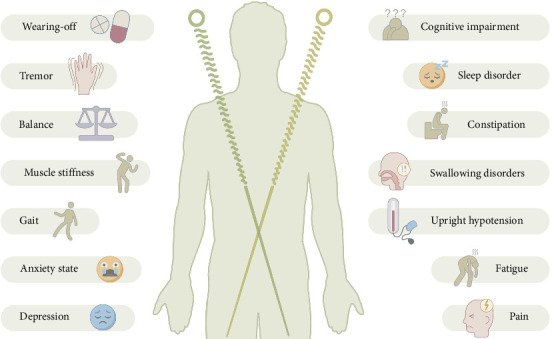
Acupuncture for PD symptoms.

**Figure 5:**
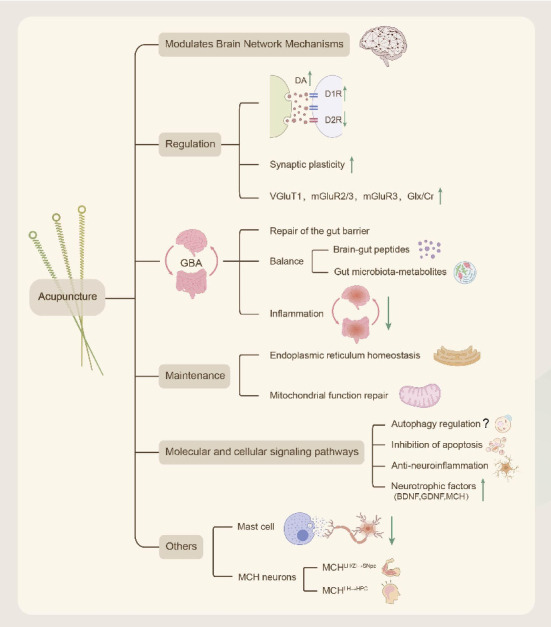
Mechanisms of acupuncture for PD.

This article summarizes the clinical efficacy of RCTs and finds that acupuncture combined with conventional treatment significantly reduces the UPDRS scores, improves gait parameters and balance function, and improves nonmotor symptoms. However, the efficacy of acupuncture in treating fatigue remains controversial. Future studies should incorporate a more rigorous, blinded design with objective biomarker validation (Figure 4). This review is the first to systematically analyze the mechanism of action of acupuncture at multiple levels, including neuromodulation (dopamine/glutamate system and brain network remodeling), GBA interaction (flora–metabolite–immunity axis), organelle homeostasis (endoplasmic reticulum–mitochondria synergy), and molecular pathways (autophagy–apoptosis balance and neuroinflammation inhibition), which represents a breakthrough from the traditional single-target research paradigm. The findings of these new mechanisms are summarized, including the revelation that acupuncture inhibits neuroinflammation and promotes the survival of dopaminergic neurons by activating the release of mediators (e.g., histamine and tryptophan-like enzymes) from local MC at acupoints, providing a new perspective on the regulation of peripheral–central immune interactions by acupuncture. Additionally, acupuncture improves PD symptoms by activating specific MCH neuron subpopulations (SNpc projections improve motor function and HPC projections restore memory function) and modulating neural circuits, which lays a theoretical foundation for precise targeted therapy (Figure 5).

However, the efficacy of acupuncture remains controversial, particularly regarding fatigue symptom improvement, which may be related to the choice of acupoints or the sensitivity of the assessment tool. This highlights the need to standardize control designs, expand the sample size of the RCT, and introduce objective biomarkers to enhance the reliability of conclusions in the future. Meanwhile, the bidirectional regulation of autophagy by acupuncture, along with the varying results regarding Nrf2 activation and mTOR inhibition, suggests that the effects of acupuncture may be influenced by different pathological stages or acupuncture stimulation parameters, warranting further investigation.

This review has the following limitations: (1) Heterogeneity of clinical evidence: The intervention protocols included in the RCTs varied widely, which may have affected the reliability of the efficacy of acupuncture. (2) Limitations of mechanistic studies: Most current mechanistic studies originate from animal models, and there is a lack of human tissue or imaging studies. (3) Lack of long-term follow-up data: Most studies did not assess the long-term effects and safety of acupuncture.

## 5. Future Expectations

Acupuncture treatment for PD should be explored in the following ways: (1) Standardization and precision research: Standardized protocols for acupuncture intervention (e.g., acupoint combinations, stimulation parameters, and treatment schedules) should be established, and individualized treatment protocols should be optimized by combining artificial intelligence (AI) and machine learning. (2) Cross-scalar mechanism analysis: Connectomics, metabolomics, and intestinal macrogene genomics should be integrated to reveal the multidimensional control network of acupuncture and moxibustion in brain immunity. (3) Translational medicine and technological innovation: Smart acupuncture devices combined with wearable sensors should be developed to provide real-time feedback on motor/nonmotor symptom improvement and improve treatment accessibility and adherence. (4) The synergistic effects of acupuncture and emerging therapies (e.g., α-syn antibodies, gene editing), especially the complementary mechanisms targeting dopaminergic and nondopaminergic pathways, should be explored. (5) Strengthening and globalization of evidence-based medicine: a multicenter, large-sample, long-cycle RCT should be designed to incorporate objective endpoints and reduce interference of the placebo effect.

## 6. Conclusion

Acupuncture improves the motor and nonmotor symptoms of PD through multiple mechanisms, including modulation of the dopamine and glutamatergic systems, regulation of the GBA, maintenance of organelle homeostasis, and inhibition of neuroinflammation/oxidative stress, reflecting the multitarget therapeutic features of acupuncture. Although some efficacy mechanisms remain to be elucidated, the current review suggests that acupuncture may be an important complementary alternative for the treatment of PD. The transformation of acupuncture from empirical to precision medicine will be further promoted through standardized clinical studies and the integration of multidisciplinary techniques in future studies.

## Figures and Tables

**Table 1:** The clinical efficacy of acupuncture in the patients with PD.

Study	Sample size (*n*)	Intervention (groups)	Acupoint selection (primary/secondary)	Procedure (needling method/duration)	Primary outcomes (scales/assessments)	Study type	Source
Wang et al. (2016)	91	Drug group: Madopar; EA1 group: 2 Hz/100 Hz + Madopar; EA2 group: 100 Hz + Madopar	Unspecified (presumed head or motor-related acupoints)	EA (continuous wave: 2 Hz/100 Hz or 100 Hz), daily treatment for 30 days, follow-up to 180 days	UPDRS, TAS scores	RCT	[14]
Lin et al. (2018)	37	Control: Cognitive training + drugs; treatment: EA at GV20 + control therapy	Primary: GV20	EA (dense-disperse wave, 80 Hz), once daily, 6 sessions/week for 6 weeks	TMT-A, TMT-B, MoCA, UPDRS-II, UPDRS-III	RCT	[15]
Li et al. (2021)	100	Control: Madopar; combined: EA + Madopar	Primary: GV20, EX-HN1, LR3, HT7, LI4, SP6, EX-HN22	EA (dense-disperse wave, 2 Hz/15 Hz), once daily, 4 weeks/course (2 courses)	5-HT, DA, BDNF levels; UPDRS, PDSS, PSQI, SAS, SDS scores	RCT	[16]
Chen et al. (2013)	114	Acupuncture: “Cranial base seven points”; drug: Madopar	Primary: Cranial base seven points	Even reinforcing-reducing technique (120 rotations/min), alternate-day sessions, 9 sessions/course (3 courses, 9 weeks)	mWS, clinical efficacy, adverse events	RCT	[17]
Xia et al. (2013)	60	Control: Madopar + Fluoxetine; treatment: EA + control therapy	Primary: GV20, EX-HN3, EX-HN1, LR3, SP6; secondary: LR8, CV17 for syndromes	EA (dense-disperse wave, 16–18 rotations/min), alternate-day sessions, 3 months/course	HAMD scores, DA concentration	RCT	[18]
Fan et al. (2022)	120	Treatment: Governor vessel three needles + hand-foot spasm needles + anti-PD drugs; control: sham acupuncture + anti-PD drugs	Governor vessel three needles (GV14, GV8, and GV4); hand-foot spasm needles (e.g., HT1, LU5, PC6)	Lifting-thrusting balanced technique, 4 sessions/week for 8 weeks	UPDRS-III, Ashworth scale, 10-m walk test, serum BDNF, and α-syn levels	RCT	[19]
Liu et al. (2018)	96	Control: Carbidopa–levodopa + placebo; treatment: EA at zusanli (ST36), Tianshu (ST25) + Carbidopa–levodopa + placebo	Primary: ST36, ST25	EA (continuous wave, 1.5 Hz), once daily (20 min/session), 7-day course	Anorectal manometry (resting pressure), constipation symptom scores, SF-36 QoL	RCT	[20]
Liu et al. (2020)	60	Control: Duloxetine; treatment: Duloxetine + acupuncture	Primary: Liver zone, Sanjiao zone, GV20, GB20, GV16, LR3	EA (160 rotations/min), once daily for 28 days	HAMD, HAMA scores, clinical efficacy	RCT	[21]
Huang et al. (2014)	40	Control: Madopar; treatment: Madopar + Fang's scalp acupuncture	Scalp zones: “Fu Xiang,” “signal,” “thinking,” “motor balance,” “memory”	Rapid insertion to periosteum (160 rotations/min), 30-min retention, 5 sessions/week for 4 weeks	UPDRS total/subitems, PSQI	RCT	[22]
Li et al. (2020)	55	Control: Conventional drugs; treatment: EA + conventional drugs	Primary: Bilateral anterior Ding–Xuan Lu line; secondary: LI11, LI4, GB34, ST36, SP6, KI3	EA (10/50 Hz), 3 sessions/week for 12 weeks	UPDRS, PDQ-39, 20-m walk time, step length	RCT	[23]
Huang et al. (2015)	120	Transcranial EA group (EA + Madopar); regular EA group (regular EA + Madopar); drug control (Madopar)	Bilateral chorea-tremor control area	Transcranial EA: 200 rotations/min, 40 min/session, 8 weeks; regular EA: no rotation, same duration	TCM syndrome scores, PD rating scales	RCT	[24]
Han et al. (2022)	96	Acu-drug group (Jiaji Panlong needling + Madopar); drug group (Madopar)	C7–L5 EX-B2	Jiaji Panlong needling: Once daily, 20 sessions/course (4 courses, 16 weeks)	UPDRS-III, UPDRS-IV, TCM syndrome scores, PDQ-39	RCT	[25]
Cai et al. (2020)	97	Treatment: Cranial base seven points + existing PD therapy; control: Escitalopram + existing PD therapy	Bilateral GB20, GB12, BL10, GV15	Alternate-day sessions, 15 sessions/course (3 courses, 3 months)	HAMD, UPDRS-I, BDI-II scores	RCT	[26]
Luo et al. (2023)	60	Control: Western drugs; treatment: Western drugs + meridian-sinew acupuncture	Primary: GV24, GV20, C4 JEX-B2, BL14; secondary: Jianqian (extra), LI10, PC7	Longitudinal/transverse needling (15° insertion), 5–6 acupoints/session, 3 sessions/week for 4 weeks	UPDRS-III, PD QoL questionnaire, HAMA, HAMD, PSQI, TCM syndrome scores	RCT	[27]
Li et al. (2016)	40	Treatment: Governor vessel-regulating acupuncture + Madopar; control: Madopar	Primary: GV24, GV20, GV16, GV9, GV8, GV14, GV4; secondary: Scalp motor areas	Balanced reinforcing-reducing technique, 30-min retention, 6 sessions/week for 8 weeks	Webster scale, total efficacy	RCT	[28]
Li et al. (2018)	60	Treatment: Governor vessel-regulating acupuncture + levodopa–benserazide; control: levodopa–benserazide	Primary: GV24, GV20, GV18, GV17, GV16	Balanced reinforcing-reducing technique, 30-min retention, once daily, 2 weeks/course (3 courses)	MoCA, MMSE scores	RCT	[29]
Liu et al. (2016)	40	Treatment: Scalp EA + levodopa–benserazide; control: levodopa–benserazide	Primary: Jiao's scalp chorea-tremor control area	EA (continuous wave, 2 Hz), 20 min/session, 3 sessions/week for 3 courses	UPDRS motor scores, electromyography (tremor frequency/amplitude)	RCT	[30]
Lu et al. (2022)	68	Treatment: Qin's scalp eight needles + levodopa–benserazide; control: levodopa–benserazide	Primary: GV20, EX-HN3, GB15, GB8, FGB20; secondary: ST36, LI10, LR3	EA (2 Hz), 30-min retention, 3 sessions/week for 8 weeks	UPDRS, HAMD, TCM QoL, TMT-A/B	RCT	[31]
Feng et al. (2023)	55	Treatment: drugs + rehabilitation + acupuncture; control: drugs + rehabilitation	Primary: GV16, GV20, GV24, EX-HN3; secondary: pharyngeal/swallowing-specific acupoints	Point-pricking (minor bleeding), balanced technique, 6 sessions/week for 4 weeks	SSA, SWAL-QOL, videofluoroscopic swallowing study (OTT, LCD), serum ALB	RCT	[32]
Jia et al. (2022)	66	Treatment: scalp + body acupuncture + drugs; control: drugs	Scalp: MS6, MS3, MS9, MS14; body: GV20, EX-HN1, GV14	Scalp: Oblique insertion (30°); body: balanced technique; 3 sessions/week for 8 weeks	UPDRS, UPDRS-III, MDRSPD, LED scores	RCT	[33]
Wu et al. (2021)	56	Treatment: conventional therapy + swallowing rehab + acupuncture; control: conventional therapy + swallowing rehab	Primary: CV23, Shanglianquan (extra), TE17; secondary: GB20, GB12, GV16, Nei Daying (extra)	Oblique insertion (60–40 mm depth), tongue pricking (minor bleeding), 5 sessions/week for 3 courses	Videofluoroscopy (OTT, SRT, PTT, LCD), BMI, serum ALB, PA, Hb	RCT	[34]
Feng et al. (2024)	66	Treatment: levodopa–benserazide + rehabilitation + “Governor vessel-balancing” acupuncture; control: levodopa–benserazide + rehabilitation	Primary: EX-HN3, GV24, GV20, GV16, GV14; secondary: SI3, BL62, LI4, LR3	Daily sessions, 6 sessions/week for 2 weeks	BBS, UPDRS-III, PDQ-39	RCT	[35]
Li et al. (2015)	120	Treatment: Madopar + acupuncture; control: Madopar	Acupoints: PC7, TE4, SI6, LI5, LI11, TE14, etc. (no primary/secondary specified)	Daily sessions for 3 months	Modified Webster scale	RCT	[36]
Wang et al. (2019)	100	Drug: levodopa–benserazide; Acu: acupuncture; combined: levodopa–benserazide + acupuncture	Unspecified	Daily sessions for 30 days	UPDRS, MoCA, MMSE, ADL	RCT	[37]
Li et al. (2024)	68	Treatment: anti-PD drugs + Midodrine + stellate ganglion acupuncture; control: anti-PD drugs + Midodrine	Primary: Bilateral ST9, stellate ganglion region	Perpendicular insertion (10–15 mm depth), no retention, daily sessions (5/week) for 2 weeks	SBP, DBP, OHQ, UPDRS, serum NE	RCT	[38]
Zhang et al. (2022)	64	Treatment: levodopa–benserazide + rehabilitation + acupuncture; control: levodopa–benserazide + rehabilitation	Primary: EX-HN1, GV16, GV26, bilateral GB20, PC6, LI4, LR3, etc.	Scalp: Subdermal insertion; body/limbs: Perpendicular insertion; 20-min retention, daily for 8 weeks	UPDRS	RCT	[39]
Bai et al. (2021)	58	Treatment: alprazolam + acupuncture; control: alprazolam	Primary: GV20, EX-HN3, GB20, EX-HN22, CV6, CV12, ST25, PC7, SP6	Scalp: Retrograde insertion; limbs: balanced technique; 40-min retention, daily for 2 weeks	PSQI, HAMA scores	RCT	[40]
Lin et al. (2018)	60	Treatment: Anti-PD drugs + Siguan acupoints (Hegu (LI4), Taichong (LR3)); control: anti-PD drugs	Primary: Bilateral LI4, LR3	Rapid insertion (1.0–1.2 cun), reducing technique, 30-min retention, daily for 4 weeks	UPDRS, WOQ-9	RCT	[41]
Zhou et al. (2014)	60	Treatment: Madopar + acupuncture (chorea-tremor control area, Siguan points, Baihui (GV20)); control: Madopar	Primary: chorea-tremor control area, Siguan points (LI4, LR3), GV20	Oblique insertion (chorea-tremor area), Baihui (GV20) posterior insertion, Siguan points perpendicular, 30-min retention, daily for 4 weeks	UPDRS	RCT	[42]
Gu et al. (2023)	103	Treatment: levodopa–benserazide + sertraline + acupuncture; control: levodopa–benserazide + sertraline	Primary: GV20, EX-HN3, GB13, PC6, HT7, GB40, PC7	Balanced technique, 60-min retention, daily sessions (weekdays only) for 3 months	UPDRS, YBCS, HAMA, HAMD, improvement rates	RCT	[43]
Zhong et al. (2023)	60	Treatment: levodopa–benserazide + pramipexole + piribedil + acupuncture; control: drugs only	Primary: TE4, LI11, SI9, KI3, SP9, BL36, etc.	Plum-blossom needle tapping followed by shallow insertion, 0.5-h retention, daily for 3 courses (15 days/course)	MDS-UPDRS-III, gait parameters (speed, turning angle), TCM symptom scores	RCT	[44]
Li et al. (2018)	78	Control: Madopar; treatment: Madopar + acupuncture	Scalp: Bilateral Dingnieqianxie line; body: LI11, GB20, LI4, KI3, LR3, GB34	Scalp EA (continuous wave, 2 Hz), 20 min/session, 3 sessions/week for 3 courses (4 weeks/course)	UPDRS, total efficacy	RCT	[45]
Liu et al. (2023)	118	Control: conventional therapy; treatment: conventional therapy + acupuncture	ST36, GV20, SP6	Warm needling (moxa 10 min) at zusanli (ST36)/Sanyinjiao (SP6), suspended moxa at Baihui (GV20), daily for 4 weeks + 3-month follow-up	FSS, PDQ-39, SDS	RCT	[46]
Jiang et al. (2020)	66	Control: levodopa; treatment: levodopa + acupuncture	ST25, ST37, ST36, CV6	Balanced technique (manipulated every 10 min), 30-min retention, 20-day course	CSS, PAC-QOL, PDQ-39	RCT	[47]
Qiao et al. (2022)	58	Control: Madopar + Pramipexole; treatment: Madopar + Pramipexole + acupuncture	ST25, ST37, ST36, CV6	Warm needling (30-min retention), daily for 20 days	CSS, PAC-QOL, SCOPA-AUT	RCT	[48]
Zhao et al. (2022)	60	Treatment: integrated acupuncture (needling + moxibustion + auricular); control: Sham (sham needling + sham moxa + sham auricular)	GV20, ST8, CV23, TE5, GB41, SP6, “Qi-regulating” points (CV12, CV10, CV6, CV4)	Needling (30-min retention) + moxibustion (2 cones/site) + auricular intradermal needles; 2 sessions/week for 12 weeks	PDSS, PSQI, total efficacy	RCT	[49]
Fan et al. (2022)	70	Real acupuncture: needling + monitoring; Sham: Sham needling + monitoring	GV24, GV29, HT7, SP6, Sishenzhen (GV21, GV19 + bilateral 1.5 cun)	Needling (30-min retention), 3 sessions/week for 8 weeks	HAM-A, UPDRS, PDQ-39, serum ACTH/CORT	Double-blind RCT	[50]
Jang et al. (2020)	26	Treatment: acupuncture (twice/week) + conventional therapy; control: conventional therapy	Unspecified (back acupoints per STRICTA)	Traditional needling, twice/week for 4 weeks	GAITRite (gait parameters), fNIRS (cerebral hemodynamics), UPDRS (walking/balance), plasma/serum neurotransmitters	Single-blind RCT	[51]
Kong et al. (2018)	40	Real: standard acupoints; Sham: nonacupoints (park sham device)	PC6, LI4, ST36, SP6, KI3, CV6	Twice/week for 5 weeks (20 min/session)	MFI-GF, UPDRS motor, PDQ-39, GDS, ESS	Patient/assessor-blinded RCT	[52]
Lei et al. (2016)	15	EA: standard acupoints + electrical stimulation; Sham: nonacupoints + no stimulation	GV20, GV14, LI4, ST36, GB34, SP6, KI3, LR3	EA (4 Hz/100 Hz), once/week for 3 weeks (30 min/session)	Wearable sensors (gait speed, stride, double support time), UPDRS, SF-12, FES-I	Double-blind RCT	[53]
Kluger et al. (2016)	94	Real: standard acupoints; sham: nonacupoints (blunt toothpick)	GV20, GV24, CV6, LI10, HT7, ST36, SP6	Twice/week for 6 weeks (30 min/session)	MFIS, PDQ-39, HADS, PDSS, ESS	Patient/assessor-blinded RCT	[54]
Wang et al. (2024)	60	EA; sham (park sham device)	Primary: GV20, CV6, LU7, SJ19; secondary: LI15, LI11, SP10, ST36	EA (2 Hz/100 Hz alternating), 30-min retention, 5 sessions/week for 4 weeks	KPPS, VAS, SWE elastography (Young's modulus, shear wave velocity), MAS, UPDRS-II/III, HAMD	Single-center RCT	[55]
Nazarova et al. (2022)	35	EA (scalp-abdominal); control: conventional drugs	GV20, GB20, CV4, CV12, ST25, ST36, SP6, LI4, ST40, LR3	EA (50/100 Hz, patient-tolerated intensity), 30-min retention, twice/week for 8 weeks	NMSS, PDSS, BSFS, PAC-QOL, UPDRS, Hoehn–Yahr stage, gut microbiota (16S rRNA)	RCT	[56]
Toosizadeh et al. (2015)	15	EA; sham	GV20, GV14, bilateral motor/sensory areas, ST36, LI4, GB34, LR3, KI3, SP6, BL40	EA (4 Hz/100 Hz), 30 min/session, once/week for 3 weeks; sham: nonacupoint shallow needling + low-intensity stimulation	COG sway ratio (ML/AP), ankle/hip coordination, UPDRS-II/III, QoL (SF-12, FES-I, VAS)	RCT	[57]
Wang et al. (2024)	60	Treatment; fire needling; control: conventional drugs	GV16, GB20, BL10, GB12, Ashi points	Fire needling (2 pricks/site, 3–5 mm depth), 3 sessions/week for 8 weeks; control: Drugs only	KPPS, VAS, UPDRS-III, PDQ-39	RCT	[58]
Yan et al. (2024)	78	Real; sham	SI Shenzhen (bilateral), GV24, GV29, LI4, LR3, SP6, HT7, ST36, BL62, KI6	Double-blind needling (real: device-assisted; sham: blunt nonpenetrating needles), 3 sessions/week for 4 weeks	PDSS, ESS, UPDRS-III, NMSS, PDQ-39	Double-blind RCT	[59]

*Note:* Acupoint: GV20: Baihui, EX-HN1: Sishencong, LR3: Taichong, HT7: Shenmen, LI4: Hegu, SP6: Sanyinjiao, EX-HN22: Anmian, EX-HN3: Yintang, CV17: Danzhong, GV14: Dazhui, GV8: Jinsuo, GV4: Mingmen, HT1: Jiquan, LU5: Chize, PC6: Neiguan, ST36: Zusanli, ST25: Tianshu, GB20: Fengchi, GV16: Fengfu, LI11: Quchi, GB34: Yanglingquan, KI3: Taixi, EX-B2: Jiaji, BL10: Tianzhu, GB12: Wangu, GV24: Shenting, GV18: Qiangjian, GB15: Toulinqi, GB8: Shuaigu, CV23: Lianquan, TE17: Yifeng, SI3: Houxi, BL62: Shenmai, PC7: Daling, TE4: Yangchi, SI6: Yanglao, LI5: Yangxi, TE14: Jianliao, ST9: Shuitu, GV26: Shuigou, CV6: Qihai, CV12: Zhongwan, GB13: Benshen, GB40: Qiuxu, SP9: Yinlingquan, BL36: Chengfu, ST8: Touwei, TE5: Waiguan, GB41: Zulinqi, CV4: Guanyuan ST37: Shangjuxu, LI10: Shousanli, LU7: Lieque, SJ19: Tinggong, LI15: Jianyu, SP10: Xuehai, ST40: Fenglong, BL40: Weizhong, KI6: Zhaohai, SF-36/SF-12: Short Form Health Survey, DA: dopamine, 5-HT: serotonin, α-syn: alpha-synuclein, NE: norepinephrine, ACTH: adrenocorticotropic hormone, CORT: cortisol, ALB: albumin, PA: prealbumin, Hb: hemoglobin.

Abbreviations: BBS = Berg Balance Scale, BDI-II = Beck Depression Inventory-II, BDNF = brain-derived neurotrophic factor, BMI = body mass index, EA = electroacupuncture, ESS = Epworth Sleepiness Scale, FES-I = Falls Efficacy Scale-International, FSS = Fatigue Severity Scale, HADS = Hospital Anxiety and Depression Scale, HAMA = Hamilton Anxiety Rating Scale, HAMD = Hamilton Depression Scale, KPPS = King's Parkinson's Pain Scale, LED = Levodopa Equivalent Dose, MAS = Modified Ashworth Scale, MDS-UPDRS = Movement Disorder Society-Sponsored Revision of UPDRS, MMSE = Mini-Mental State Examination, MoCA = Montreal Cognitive Assessment, mWS = Modified Webster Scale, NMSS = Non-Motor Symptoms Scale, PAC-QOL = Patient Assessment of Constipation-Quality of Life, PDQ-39 = Parkinson's Disease Questionnaire-39, PDSS = Parkinson's Disease Sleep Scale, PSQI = Pittsburgh Sleep Quality Index, QoL = Quality of Life, SAS/SDS = Self-Rating Anxiety/Depression Scale, SCOPA-AUT = Scales for Outcomes in Parkinson's Disease-Autonomic, SWAL-QOL = Swallowing Quality of Life Questionnaire, TAS = Tension Assessment Scale, TCM = traditional Chinese medicine, TMT-A/B = Trail Making Test Part A/B, UPDRS = Unified Parkinson's Disease Rating Scale, VAS = Visual Analog Scale.

**Table 2:** Acupuncture treatment for Parkinson's disease based on animal models.

Study	PD model	Intervention	Acupoint	Behavioral measurements	Biochemical measurements
Jang et al. [60]	MPTP mice	MA	GB34, ST36	Cylinder test: the number of wall touches↑; rotarod test: latency time↑; akinesia test: latency time↓; open field test: the total distance↑, central distance↑, the ratio of central/total distance↑, the number of crossings in the central zone↑	DAergic fibers and neurons in the ST and SN↑, TH-positive neurons in the ST and SN↑, TH in the ST and SN↑, Bax↓, Bcl-2↑, GFAP↓, Iba-1↓, NF-*κ*B↓, TNF-*α*↓, the Chao1 index↑, the number of observed OTUs↑, the Shannon index↑, Rikenellaceae↑, Vallitaleaceae↑, Alistipes↑, Vallitalea↑, Lachnoclostridium↑, Pseudoclostridium↑, Bacteroides xylanolyticus↑, Vallitalea pronyensis↑, Clostridium aerotolerans↑, Pseudoclostridium thermosuccinogenes↑, Roseburia faecis↑, holdemania↓, frisingicoccus↓, aestuariispira↓, sporobacter↓, rhodospirillum↓, Bifidobacterium↓, turicibacter↓, marvinbryantia↓, desulfovibrio↓, phascolarctobacterium↓, erysipelatoclostridium↓, butyricimonas↑, gracilibacter↑, phocea↑, desulfitobacterium↑, oscillibacter↑, acutalibacter↑, flintibacter↑, photosynthesis↑, glutathione metabolism↑, tetracycline biosynthesis↑, photosynthetic proteins↑, methane metabolism↓, drug metabolism↓
Han et al. [61]	MPTP mice	EA	ST36, GV20	The rotarod test: the performance time and the rod speed↑ pole test: total time↑	TH in the SN↑, the Simpson index↓, intestinal microbial alpha diversity↑, the abundance of Erysipelotrichaceae↓, IL-6 and TNF-α in the SN↓
Yu et al. [62]	6-OHDA rats	EA	CV-12, CV-6, LR-3, ST-36	AIMs scores↓, rotational behavior: the net number of rotations↓, the cylinder test:↑, exploratory behavior↑, the spontaneous alternation ratio↑, open field test: the immobility time↓, the number of struggles↑	TH-positive cells in the SN↑, apoptotic cells↓, cleaved caspase-3 and cleaved PARP↓, TNF-α and IL-1β↓, COX-2 at mRNA↓, MDA↓, SOD↑GSH↑, NPY protein expression↑, CCK, SST, GAS in the serum↑, PYY in the serum↓
Tian et al. [63]	MPTP mice	MA	GB34	Rotarod test: the ORP scores↑	α-syn in the SNpc↓, autophagosome accumulation↓, LC3-II↓, LAMP1↓, DA neurons↑, density of TH-positive neurons in the SNpc and ST↑, DA concentration↑, synaptophysin protein↑
Ning et al. [64]	6-OHDA rats	MA	GV20, GV29, LI4, LR3, DU4	Open field test: horizontal activity distance↑, vertical activities↑, forced swimming test: immobility time of forced swimming↓, sucrose preference test: the sugar water preference rate↑	DA and 5-HT in the midbrain↑, α-syn in the striatum↓, Beclin-1↓, p62↑, autophagosomes↓, p-p70s6K↑, synapsin I↑ and PSD95↑ in the prefrontal cortex
Lee et al. [65]	MPTP mice	MA	Acu1: GB34; Acu2: SI3	Pole test: descending times (GB34)↓	TH-positive neurons in the SN (GB34)↑, caspase-3 in the ST (GB34)↓, DJ-1 in the ST (GB34)↑, SOD activity (GB34)↑, CAT activity (GB34)↑
Deng et al. [66]	GFAPtTA/tetO-α-syn (A53T) double transgenic mice	EA	ST36,SP6	EA reduced loss of body weight, delayed paralysis onset, and increased lifespan in A53T mice; open field test: the movement distance↑, rotarod test: the motor coordination↑; grip strength test: the mean grip force in the fore- and hindlimbs↑; catwalk gait analysis: stride length in the left-fore, left-hind, and right-hind paw↑, interlimb coordination measured: Regularity index↑	α-Syn in the midbrain and ST↓, GFAP-immunoreactive astrocytes↓, the expression of GFAP in the SN↓, the intensity of Iba-1 immunostaining and the clusters of activated microglia↓, TNF-α in the SN and striatum↓, IL-1β in the striatum↓, TH-positive neurons in the SNpc↑, DA content↑, Nrf2 protein levels in the midbrain and ST↑, the Nrf2 mRNA level in the midbrain↑, HO-1 protein expression in the midbrain and striatum↑, GCLM expression in the midbrain↑, HO-1 mRNA expression in the midbrain↑, GCLM mRNA expression in the midbrain↑
Lv et al. [67]	MPTP mice	EA	ST36, SP6	Open field test: the distance in vertical activity↑	CAT levels in the ST↑, MDA↓, SOD levels in the ST↑, DNP-modified protein levels in the striatum↓, the intensity of Iba1 immunostaining in the midbrain↓, TNF-α, IL-6, and IL-1β↓, the mean OD value of GFAP-immunoreactive cells in the midbrain↓, the mean OD value of GFAP-immunostaining cells in the striatum↓, TH fibers in the ST↑, TH protein expression in the midbrain↑, TH protein expression in the striatum↑, the expression of DAT ↑, VMAT2 in the ST↑, hPAP in the midbrain and ST↑, NQO1 in the midbrain and ST↑, Nrf2, HO-1, and NQO1 in the ST
Wang et al. [68]	Rotenone rats	EA	GV16, LR3	—	BDNF mRNA expression in the SN↑, GDNF mRNA expression in the SN↑
Pak et al. [69]	MPTP mice	EA	GV20, GV14,	The rotarod test: rotarod latency↑, the catwalk test: right front paw; left front paw and left-hind paw↑	The mean IOD in the ST and number of TH-positive cells in the SN↑, levels of free mBDNF and mGDNF in both the cerebrum and midbrain↑, DA in the ipsilateral (lesioned) SN and ST↑, pTrkB expression (i.e., mBDNF receptor expression) ↑GFRα-1 expression (i.e., GDNF receptor expression)↑, pCREB↑, pCREB/TH double-positive cells↑, pAkt/TH-positive cells↑, Pitx3/TH double-positive cells↑
Zhao et al. [70]	MPTP mice	EA	GV20, GV29	Rotarod test: latency time↑; tail suspension test: the immobility time↓	TH in the midbrain↑, BDNF^+^ cells↑, pAkt↑, pERK↑, functional TrkB FL↑, the ratio of TrkB FL/TrkB T1↑
Wang et al. [71]	6-OHDA rats	EA	GV 20, GV 14	APO-induced rotation test: APO-induced rotation↓, rotarod test: Latency time↑	VGluT1 levels in the STN↑
Li et al. [72]	6-OHDA rats	EA	GV 20, GV 14	Open field test: FP move time (A) and FP distance↑; rotarod test: Latency time↑	Striatal LTP in dorsolateral striatum↑, NR2B↑, striatal spine density↑, the striatal dendritic arborization↑, striatal Glu↓, VGLUT1 in the ST↓
Jia et al. [73]	6-OHDA rats	EA	GV 20, GV 14	APO-induced rotation↓, rotarod test: latency time↑, the total movement distance↑, the total movement time↑, the mean velocity↑	Glu contents in the cortex and ST↓, mGluR3 mRNA expression↑, mGluR2 mRNA expression↓, mGluR2/3 proteins in the ST↑
Li et al. [74]	6-OHDA rats	EA	GV 20, GV 14	Open field test: FP move time↑, FP distance↑, rotarod test: Latency time↑	The Glx/Cr ratio in the ST↓
Oh et al. [75]	MPTP mice	MA	GB34	Cylinder test: the number of wall touches↑, rotarod test: latency time↑	DAergic fibers↑, TH-positive cells in ST and SN↑, the expressions of c-Fos in CA3↑, the expressions of c-Fos in DG↑, the expressions of c-Fos in LH↑, the expressions of c-Fos in SoL↑, the expressions of c-Fos in DLS↓, the expressions of c-Fos in DMS↓, the expressions of c-Fos in SNpc↓, the expressions of c-Fos in Gp↓
Wang et al. [76]	6-OHDA rats	MA	LR3, LI4, GV20, GB20, DU16, ST36	Open field test: number of crossing↑, number of rearing↑, the central time stopped↓; rotarod test: Latency time↑, forelimb akinesia test: initiation time↓	DA and 5-HT levels in the ST↑, α-syn in the midbrain↓, GRP78 and CHOP in the midbrain↑, the expression of p-IRE1 and p-XBP1 in the ST↑
Li et al. [77]	6-OHDA rats	MA	CV12, CV4, ST25	—	TH-positive neuron in the SN↑, TNFα, IL-1β in the duodenum and serum↓, deposition of Fe in the SN↓, the mRNA ratio of DMT1/Fpn1 in the SN which differed both significantly from the untreated and Madopar-treated groups, α-syn in the duodenum↓
Yeo and Lim [78]	MPTP mice	MA	GB34, LR3	—	TH-positive neuron in the ST and SNpc↑, α-syn in the SN↓, SGK1↑
Gao et al. (2013) [79]	6-OHDA rats	EA	GB34, ST36, SP6	APO-induced rotation: the numbers of rotations↓	TH-positive neuron in the SN↑, DAT in the ST and SN↑, D1R in the ST↑, D2R in the ST↓
Rui et al. [80]	A53T mutant mice	MA	GB34	The rotarod test: latency time↑	TH-positive neurons in the SN and DAergic fibers in ST↑, pro-MCH in the hypothalamus, MCH in the SN↑
Geng et al. [81]	MPTP mice	EA	The chorea trembling control area	Rotation tests: the residence time on the rotating rod↑, the forelimb grip strength↑	TH-positive neurons, PARKIN, PINK1, α-syn↑, Nissl bodies in the cytoplasm↑, SOD↑ GSH-Px↑, MDA and TNF-α, IL-6 and IL-1β↓, the expression of TRPC1↑, Ca2+↑, PARKIN↑, PINK1↑, PGC-1α↑, mfn2↑, Drp1↓, mitochondrial membrane potential↑, SIRT1↑, p-AMPK proteins↑
Ma et al. [82]	ROT mice	EA	ST25, DU24, ST37, LI11	Open field tests: the total distance↑, the total duration↑ rotarod tests: latency time↑	TH in SN↑, Iba-1↓, α-syn in the colon and the brain↓, ChAT in the colon↑, TH in the colon↑, nNOS in the colon↑, IL-1β in the colon↓, IL-6 in the colon↓, TNF-α in the colon↓, CD^3+^↓, ZO-1 in the colon↑, occludin in the colon↑
Hu et al. [83]	ROT rats	EA	ST25	Rotarod tests: Latency time↑; pole test: time to climb down↓	TH-positive neurons↑; Lactobacillus↑, Dubosiella↑, Bifidobacterium↑, Escherichia–Shigella↓, Morganella↓; HHN in SNpc↓, HNDE in SNpc↓, HNE in SNpc↓, LPS↓, IL-6, and TNF-α↓, IFN-γ↓, pPE↑, LPE↑
Lin et al. [84]	MPTP mice and MPP^+^ rats	EA	GB3, LR3	Rotarod tests: Latency time↑ (MPTP mice); APO-induced rotation test: turning behavior (C) and locomotor activity↓(MPP^+^)	DA in the SN↑(MPTP mice), TH in the SN↑(MPTP mice), TH-positive neurons in the SN(MPP^+^), DA in the ST↑(MPP^+^)BDNF↑(MPP^+^), Bcl-2↑(MPP^+^), pAkt↑(MPP^+^)
Guo et al. [85]	MPTP mice	EA	GV16, LR3, ST36	Open field test: the total distance↑, moving duration↑, resting duration, average speed↑, center distance↑, and zone crossing ↑	WBC↑, RBC↑, HGB↑, MCHC↑, LYM↑, Claudin-1 in colon↑, TH in colon↑, BDNF in colon↑, DAT in colon↑, GDNF in colon↑, and α-syn↓, Bax in colon↓, Bcl-2 in colon↑, caspase-3 in colon↓, IL-1β, IL-18, IL-6, and TNF-α in the colon↓; IL-10 and TGF-β in the colon↑, NLRP3 and caspase-1 in colon↓
Oh et al. [86]	MPTP mice	MA	GB34	Rotarod test: latency to fall↑, cylinder test: forelimb use↑, Y-maze: spontaneous alternation↑, NOR: Recognition index test↑	TH + Neurons↑, striatal DA↑, Neuroinflammation↓, MCH Activity↑, oxidative stress↓, BDNF↑
Jiang et al. [87]	6-OHDA rats	EA	GV14, GV20	Open field test: total distance↑, movement time↑, rotarod test: latency to fall↑	Corticostriatal Glu↓, beta oscillations (25–40 Hz)↓, synaptic plasticity↑, LFP coherence↓
Zhang et al. [88]	MPTP mice	EA	GV20, LR3	Rotarod test: time stayed on the rod↑, pole test: descent time↓	TH-positive neurons↑, α-synuclein expression↓, Atg7↑, LC3II↑, p62↓, nuclear Nrf2↑, Keap1↓
Zou et al. [89]	MPTP mice	EA	Not explicitly mentioned	Rotarod test: motor performance↑, pole test: climbing time↓, EPM: open-arm entries↑, anxiety index↓; open field test: total distance traveled↑, time in central zone↑	SGLT1 levels↓, TRPC1↑, Ca^2+^ concentrations↑, PI3K/AKT pathway activation↓, IL-1β↓, IL-6↓, TNF-α↓, glucose uptake↑, lactate levels↓
Oh et al. [90]	MPTP mice	MA	GB34	Cylinder test: forelimb touches↑, rotarod test: latency to fall↑, pole test: time to descend↓, elevated plus maze: open-arm exploration↑, anxiety index↓, open field test: total distance↑, central zone activity↑	TH expression↑, GFAP (astrocytes)↓, Iba-1 (microglia)↓, IL-1β↓, IL-6↓, TNF-α↓, mast cell number↑, degranulation ratio↑, HDC/chymase/tryptase release↑

*Note:* MPTP: 1-methyl-4-phenyl-1,2,3,6-tetrahydropyridine, MA: acupuncture; GB34: *Yanglingquan*, ST36: *Zusanli*, DAergic: Dopaminergic; ST: striatum, SN: substantia nigra, Bax: Bcl-2-associated X protein; TNF-*α*: tumor necrosis factor-*α*; Bcl-2: B-cell lymphoma-2; Iba-1: ionized calcium-binding adaptor molecule-1; NF-*κ*B: nuclear factor kappa-B; GV20: *Baihui*; IL-6: interleukin-6; 6-OHDA: 6-hydroxydopamine; CV-12: *Zhongwan*; CV-6: *Qihai*; LR-3: *Taichong*; IL-1β: interleukin-1β; COX-2: cyclooxygenase; MDA: malondialdehyde; SOD: superoxide dismutase; GSH: glutathione; NPY: neuropeptide Y; CCK: cholecystokinin; SST: somatostatin; GAS: gastrin; PYY: Peptide YY; Atg: antithymocyte globulin; LC3-II: microtubule-associated protein 1 light chain 3 II; DA: dopamine; GV29: *YinTang*; LI4: *Hegu*; DU4: *MingMen*; 5-HT: 5-hydroxytryptamine; p62: p62/SQSTM1; p70s6K: 70-kDa ribosomal protein S6 kinase; PSD95: postsynaptic density protein-95; SI3: Houxi; CAT: catalase; SP6: Sanyinjiao; DNP: dinitrophenyl; ROT: rotenone; DAT: dopamine transporter; Nrf2: nuclear factor erythroid 2–related factor; HO-1: heme oxygenase-1; GV16: *Fengfu*; pAkt: phosphorylated Akt; pERK: phosphorylated extracellular-regulated protein kinase; TrkB FL: full-length TrkB; TrkB T1: splicing truncated isoforms TrkB; BrdU: 5-bromo-2-deoxyuridine; DCX: doublecortin; BL60: *Kunlun*; APO: apomorphine; VGluT1: vesicular glutamate transporter 1; NR2B: N-methyl-D-aspartic acid receptor; Glu: glutamate; mGluR: metabotropic glutamate receptor; mGluR2/3: presynaptic group II mGluRs; Glx: glutamate + glutamine; Cr: creatine; CA3: field CA3 of hippocampus; SoL: nucleus of the solitary tract; DLS: dorsolateral striatum; DMS: dorsomedial striatum; Gp: globus pallidus; CHOP: homologous protein, p-IRE1: phosphorylated inositol-requiring enzyme 1; p-XBP1: phosphorylated X-box binding protein 1; CV4: *Guanyuan*; ST25: *Tianshu*; Fpn1: Ferroportin 1; SGK1: serum- and glucocorticoid-dependent kinase 1; D1R: dopamine type receptor 1; D2R: dopamine type receptor 2; GSH-Px: plasma glutathione peroxidase; PGC-1*α*: peroxisome proliferator-activated receptor gamma coactivator 1 alpha; mfn2: mitofusin-2; Drp1: dynamin-related protein 1; SIRT1: sirtuin 1; DU24: *ShenTing*; ST37: *ShangJuXu*; LI11: *QuChi*; Chat: choline acetyltransferase; ZO-1: zona occludens-1; HHN: 4-hydroxy-2(E)-hexenal; HNDE: 4-hydroxy-2(E)-nondinenal; HNE: 4-hydroxy-2(E)-nonenal; LPS: lipopolysaccharide; MPP^+^: 1-methyl-4-phenylpyridinium; pPE: plasmenylethanolamine; LPE: lysoglycerophospholipid.

Abbreviations: AIM: abnormal involuntary movements; AMPK: AMP-activated protein kinase; BDNF: brain-derived neurotrophic factor; DG: dentate gyrus; DMT1: divalent metal transporter 1; EA: electroacupuncture; FP: floor plane; GDNF: glial cell-derived neurotrophic factor; GFAP: glial fibrillary acidic protein; GRP78: glucose regulatory protein 78; HGB: hemoglobin; hPAP: human placental alkaline phosphatase; IOD: integral optical density; LAMP1: lysosome-associated membrane protein 1; LH: lateral hypothalamus; LTP: long-term potentiation; LYM: lymphocyte; MCH: melanin-concentrating hormone; MCHC: mean corpuscular hemoglobin concentrations; NLRP3: NOD-like receptor 3; nNOS: neuronal nitric oxide synthase; NQO1: NADPH quinone oxidoreductase 1; OD: optical density; OTUs: operational taxonomic units; PARP: poly(ADP-ribose) polymerase; RBC: red blood cell; SNpc: substantia nigra pars compacta; TH: tyrosine hydroxylase; TRPC1: transient receptor potential canonical 1; VMAT2: vesicular monoamine transporter 2; WBC: white blood cell.

## Data Availability

Data sharing not applicable to this article as no datasets were generated or analyzed during the current study.

## Supporting Information

Supporting Information 1 1. Supporting Methods S1: Search terms and basic search strategy.

Supporting Information 2 2. Table S1: Cochrane Risk of Bias Assessment (RoB 2.0).
