# Supplementary material for: Neuroprotective Mechanisms and Clinical Evidence for Acupuncture in Parkinson's Disease: A Systematic Review
Source: Parkinsons Dis. 2025 May 11;2025:9739567. doi: 10.1155/padi/9739567 (PMC12086037; doi:10.1155/padi/9739567)
Supplement: Supporting Information 1 — 1. Supporting Methods S1: Search terms and basic search strategy. [file 9739567.f1.docx]

**Supplementary Methods S1. Search terms and basic search strategy**

PubMed：

(("Acupuncture"[Mesh] OR "Acupuncture Therapy"[Mesh] OR "Electroacupuncture"[Mesh] OR acupuncture[Title/Abstract] OR electroacupuncture[Title/Abstract])) AND (("Parkinson Disease"[Mesh] OR "Parkinsonian Disorders"[Mesh] OR "Parkinson’s disease"[Title/Abstract] OR "Parkinson disease"[Title/Abstract] OR parkinson*[Title/Abstract])) AND (("clinical trial"[Publication Type] OR "randomized controlled trial"[Publication Type] OR "RCT"[Title/Abstract]) OR ("Mechanisms of Action"[Mesh] OR "Neuroprotective Agents"[Mesh] OR "Oxidative Stress"[Mesh] OR "Signal Transduction"[Mesh] OR "Apoptosis"[Mesh] OR mechanism*[Title/Abstract] OR pathway*[Title/Abstract] OR neuroprotect*[Title/Abstract]))
