# Supplementary material for: Neuroprotective Mechanisms and Clinical Evidence for Acupuncture in Parkinson's Disease: A Systematic Review
Source: Parkinsons Dis. 2025 May 11;2025:9739567. doi: 10.1155/padi/9739567 (PMC12086037; doi:10.1155/padi/9739567)
Supplement: Supporting Information 2 — 2. Table S1: Cochrane Risk of Bias Assessment (RoB 2.0). [file 9739567.f2.docx]

### **Table S1. **Cochrane Risk of Bias Assessment（RoB 2.0）****

| **Study** | **Random sequence generation** | **allocation concealment** | **Blinding of participants and personnel** | **Blinding of outcome assessment** | **Incomplete Outcome Data** | **Selective reporting** | **Other Bias** | **Source** |
| --- | --- | --- | --- | --- | --- | --- | --- | --- |
| Wang XY et al. (2016) | **low** | **unclear** | **high** (EA cannot be blinded) | **low** | **low** | **low** | **unclear** | (26) |
| Lin ZC et al. (2018) | **low** | **unclear** | **high** (EA cannot be blinded) | **low** | **low** | **low** | **low** | (39) |
| Li L et al. (2021) | **low** | **unclear** | **high** (EA cannot be blinded) | **unclear** | **low** | **low** | **unclear** | (45) |
| Chen F et al. (2013) | **low** | **unclear** | **high** (Acupuncture cannot be blinded) | **unclear** | **low** | **low** | **unclear** | (14) |
| Xia Y et al. (2013) | **low** | **unclear** | **high**(EA cannot be blinded) | **low** | **low** | **low** | **unclear** | (36) |
| Fan JQ et al. -1 (2022) | **low** | **low** | **low** | **unclear** | **low** | **low** | **low** | (27) |
| Liu AG et al. (2018) | **low** | **unclear** | **high** (No blinding) | **high** (Not mentioned) | **low** | **low** | **High (**Placebo effect not controlled) | (50) |
| Liu LF et al. (2020) | **high**(Method not described) | **high** (Not described) | **high**(Blinding impossible) | **high** (Not mentioned) | **low** | **low** | **High (**Large intervention difference between groups**)** | (35) |
| Huang N et al. (2014) | **high**("Randomized" only mentioned) | **high** (Not described) | **high** (No placebo control) | **high** (Not mentioned) | **low** | **low** | **High (**Small sample size, no long-term follow-up**)** | (41) |
| Li KS et al. (2020) | **low** | **high** (Not described) | **high** (Blinding impossible) | **high** (Not mentioned) | **low** | **low** | **unclear** | (29) |
| Huang L et al. (2015) | **low** | **unclear** | **high** (EA cannot be blinded) | **unclear** | **low** | **low** | **unclear** | (15) |
| Han L et al. (2022) | **low** | **unclear** | **high**(Acupuncture cannot be blinded) | **low** | **low** | **low** | **low** | (22) |
| Cai XH et al. (2020) | **low** | **unclear** | **high**(Acupuncture cannot be blinded) | **low** | **low** | **low** | **unclear** | (32) |
| Luo WP et al. (2023) | **low** | **unclear** | **high**(Acupuncture cannot be blinded) | **unclear** | **low** | **low** | **low** | (42) |
| Li HY et al. (2016) | **low** | **high** (Not described) | **high** (Blinding impossible) | **high** (Not mentioned) | **low** | **low** | **low** | (17) |
| Li YH et al. (2018) | **high** (Random method unclear) | **high** (Not described) | **high** (Blinding impossible) | **high**(Outcome assessors unblinded) | **low** | **low** | **High (**Small sample size**)** | (38) |
| Liu XT et al. (2016) | **high**(Random method unclear) | **high** (Not described) | **high** (Blinding impossible) | **high**(Not mentioned) | **high** (Not described) | **low** | **high (**Small sample size**)** | (23) |
| Lu Z et al. (2022) | **low** | **unclear** | **high**(Acupuncture cannot be blinded) | **low** | **low** | **low** | **low** | (34) |
| Feng WX et al. (2023) | **low** | **high** (Not described) | **high** (Blinding impossible) | **high**(Not mentioned) | **low** | **low** | **high (**Small sample size**)** | (53) |
| Jia YB et al. (2022) | **low** | **low** | **high** (Blinding impossible) | **low** | **low** | **low** | **low** | (47) |
| Wu MX et al. (2021) | **low** | **high** (Not described) | **high** (Blinding impossible) | **high**(Not mentioned) | **low** | **low** | **high (**Small sample size**)** | (52) |
| Feng WX et al. (2024) | **low** | **high** (Not described) | **high** (Blinding impossible) | **high**(Not mentioned) | **low** | **low** | **high**(Potential variation in needling techniques) | (25) |
| Li YL et al. (2015) | **high** (Random method unclear) | **high** (Not described) | **high** (Blinding impossible) | **high**(Not mentioned) | **low** | **low** | **high** (Large sample but center differences uncontrolled) | (18) |
| Wang Z et al. (2019) | **high** (Random method unclear) | **high** (Not described) | **high** (Blinding impossible) | **high**(Not mentioned) | **low** | **low** | **high** (Small sample size) | (19) |
| Li MM et al. (2024) | **low** | **low** | **high** (No blinding) | **low** | **low** | **low** | **low** | (54) |
| Zhang JB et al. (2022) | **low** | **unclear** | **high** (No blinding) | **high**(Not mentioned) | **low** | **low** | **high** (Needling specificity) | (20) |
| Bai Y et al. (2021) | **high** (Grouped by visit order) | **high**(No concealment) | **high** (No blinding) | **high**(Not mentioned) | **low** | **low** | **high** (Small sample size) | (43) |
| Lin D et al. (2018) | **low** | **High** (Not described) | **high** (Blinding impossible) | **High** (Not mentioned) | **low** | **low** | **high** (Small sample size) | (48) |
| Zhou WH et al. (2014) | **low** | **low** | **high** (No blinding) | **High** (Not mentioned) | **low** | **low** | **high** (Small sample size) | (21) |
| Gu J et al. (2023) | **low** | **unclear** | **high** (No blinding) | **High** (Not mentioned) | **low** | **low** | **high** (No pure acupuncture group) | (33) |
| Zhong Y et al. (2023) | **high** (Grouped by treatment) | **high**(Not described) | **high** (No blinding) | **High** (Not mentioned) | **low** | **low** | **high** (Simplistic grouping) | (28) |
| Li GS et al. (2018) | **low** | **unclear** | **high** (No blinding) | **High** (Not mentioned) | **low** | **low** | **high** (Small sample size) | (16) |
| Liu AG et al. (2023) | **low** | **unclear** | **high** (No blinding) | **High** (Not mentioned) | **low** | **low** | **high** (Short follow-up) | (55) |
| Jiang L et al. (2020) | **low** | **unclear** | **high** (No blinding) | **high** (Not mentioned) | **low** | **low** | **high** (Small sample size) | (51) |
| Qiao F et al. (2022) | **high** (Grouped by treatment) | **high**(Not described) | **high** (No blinding) | **high** (Not mentioned) | **low** | **low** | **high** (Simplistic grouping) | (49) |
| Zhao X et al. (2022) | **low** | **unclear** | **high** (Sham control, blinding unclear) | **high** (Assessor blinding unclear) | **low** | **low** | **high** (No long-term follow-up) | (44) |
| Fan JQ et al.-2 (2022) | **low** | **high**(Not described) | **low** | **low** | **low** | **low** | **unclear** | (37) |
| Jang JH et al. (2020) | **low** | **high**(Not described) | **high** (Control group unblinded) | **high** (Partial assessors unblinded) | **low** | **unclear** | **high** (Small sample size + short intervention) | (31) |
| Kong KH et al. (2018) | **low** | **low** | **low** | **high** (Partial assessors unblinded) | **low** | **low** | **high** (Small sample size) | (56) |
| Lei H et al. (2016) | **high** (Lottery method) | **high**(Not described) | **low** | **low** | **low** | **low** | **unclear** | (30) |
| Kluger et al. (2016) | **low** | **high**(Not described) | **low** | **low** | **low** | **low** | **high** (Participant education may affect outcomes) | (57) |
| Wang SS et al. (2024) | **low** | **low** | **high**(Partial participant blinding; personnel unblinded) | **unclear** | **low** | **low** | **High (**Single-center, small sample**)** | (58) |
| Nazarova L et al. (2022) | **high**(Coin toss randomization) | **high**(Not described) | **high**(Participants/personnel unblinded) | **High** (Assessors unblinded) | **unclear** | **unclear** | **High (**Single-center, small sample ; diet confounders unaddressed**)** | (46) |
| Toosizadeh N et al. (2015) | **high**(2:1 allocation; randomization unclear) | **high**(Not described) | **high**(Partial blinding; personnel unblinded) | **High** (Assessors unblinded) | **High** (Attrition unclear; n=15) | **High** (Secondary outcomes incomplete) | **High (**Small sample; non-multicenter; confounders uncontrolled**)** | (24) |
| Wang M et al. (2024) | **low** | **low** | **high**(Participants/personnel unblinded) | **low** | **low** | **low** | **High (**Single-center; small sample; no sham control**)** | (59) |
| Yan M et al. (2024) | **low** | **low** | **unclear** | **low** | **low** | **low** | **High (**Short follow-up; homogeneous cultural background**)** | (40) |
